# The Efficacy of Currently Licensed Biologics for Treatment of Ulcerative Colitis: A Literature Review

**DOI:** 10.7759/cureus.37609

**Published:** 2023-04-15

**Authors:** Humza Awan, Urooj Fatima, Ryan Eaw, Naomi Knox, Laith Alrubaiy

**Affiliations:** 1 Department of Metabolism, Digestion and Reproduction, Imperial College London, London, GBR; 2 Gastroenterology, Swansea University Medical School, Swansea, GBR

**Keywords:** infliximab biosimilar, ustekinumab, vedolizumab, golimumab, adalimumab (humira), clinical trial & pharmacovigilance, ulcerative colitis (uc)

## Abstract

Biologics have been emerging as promising therapies in ulcerative colitis (UC) patients who are refractory to conventional medical treatment. This literature review aims to appraise the existing evidence on the efficacy and safety of NICE approved biological therapies, of which there are currently five licensed drugs, available for the treatment of UC in adults.

An initial search was performed using National Institute of Clinical Excellence (NICE) guidelines. A further literature search of EMBASE, MEDLINE, Science Direct and Cochrane Library databases was done, resulting in a total of 62 studies being included in this review. Recent and seminal papers were included. Inclusion criteria for this review were adult participants and English papers only.

In most studies, anti-tumour necrosis factor ɑ (TNFɑ) naïve patients were found to have improved clinical outcomes. Infliximab was found to be highly effective in inducing short-term clinical response, clinical remission as well as mucosal healing. However, loss of response was common and dose escalation was often required for achievement of long-term efficacy. Adalimumab was found to have both short-term and long-term efficacy which was also supported by real-world data. Golimumab was shown to have comparable efficacy and safety profiles to other biologics, although lack of therapeutic dose monitoring and loss of response is a barrier to optimising golimumab treatment efficacy. Vedolizumab was shown to have higher clinical remission rates when compared to adalimumab in a head-to-head trial, and the most cost-effective biologic when calculating quality-adjusted life years. Ustekinumab was found to significantly improve clinical remission rates in UC patients who were previously unresponsive to other biological treatments. However, as this is a newly licensed drug, there is limited literature currently available.

Further, head-to-head studies are required to help determine the optimal treatment for patients with UC. With patents expiring, the development of biosimilars will help to reduce costs and increase the availability of these drugs to patients.

## Introduction and background

Ulcerative colitis (UC) is a chronic, inflammatory disease, characterised by diarrhoea, rectal bleeding, abdominal pain as well as urgency and tenesmus [1]. These can all greatly impact daily functioning, reducing quality of life (QOL). The therapeutic aims of treatment include the induction and maintenance of remission as well as management of symptoms [2]. The National Institute for Health and Care Excellence (NICE) recommends use of biologic agents in patients with moderate-to-severe UC in whom conventional therapy such as corticosteroids, aminosalicylates or immunomodulators is contraindicated or has failed to induce a response [2-4].

**Figure 1:**
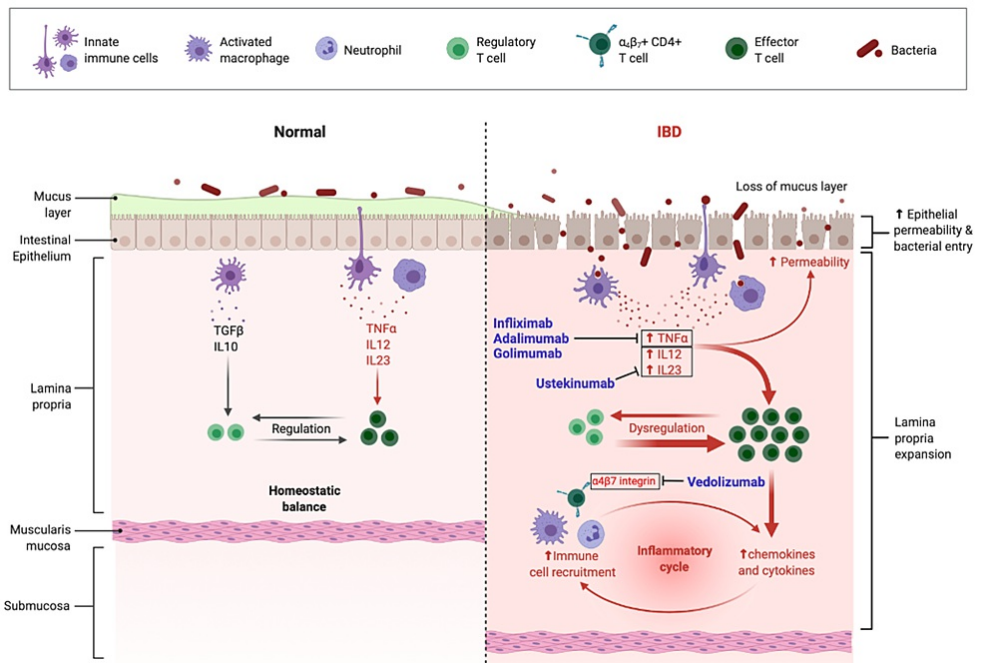
A visual representation of the mechanism of action of the NICE approved biologics. The cytokine TNFα plays a major role in the mediation of systemic inflammation in ulcerative colitis (UC). Infliximab, adalimumab and golimumab are monoclonal antibodies belonging to the class of anti-TNFα drugs [5]. They bind with a high affinity to TNFα, preventing it from binding to its receptor, thus neutralising its biologic activity and limiting inflammation [5]. Vedolizumab is another monoclonal antibody belonging to the class of anti-integrin drugs [5]. It primarily works by targeting and blocking α4β7 integrin [5]. This interferes with the migration of leukocytes to sites of inflammation and prevents further perpetuation of the inflammatory cycle [5]. Ustekinumab is an immunoglobulin (Ig)G antibody belonging to the class of anti-interleukin (IL)-12/23 drugs [5]. The cytokines IL-12 and IL-23 are both upregulated in UC [5]. Ustekinumab binds to the p40 protein subunit present in these cytokines and blocks their activity [5]. The image is created using Biorender.com.

The currently approved biologics for UC include infliximab, adalimumab and golimumab (anti-tumour necrosis factor α (TNFα) agents), vedolizumab (anti-integrin) and ustekinumab (anti-interleukin {IL}-12/23) [5]. A detailed overview of the mechanisms of the licensed biologics is outlined in Figure 1.

This literature review aims to appraise the existing evidence on the efficacy and safety of the NICE approved biologics available for the treatment of UC in adults.

## Review

Methodology

An initial search was conducted using NICE guidelines to identify important trials in the licensing of biologics for use in the UK. The keywords ‘UC’, ‘clinical trial’, ‘infliximab’, ‘adalimumab’, ‘golimumab’, ‘vedolizumab’ and ‘ustekinumab’ were used in a literature search of EMBASE, MEDLINE, Science Direct and Cochrane Library databases.

**Figure 2:**
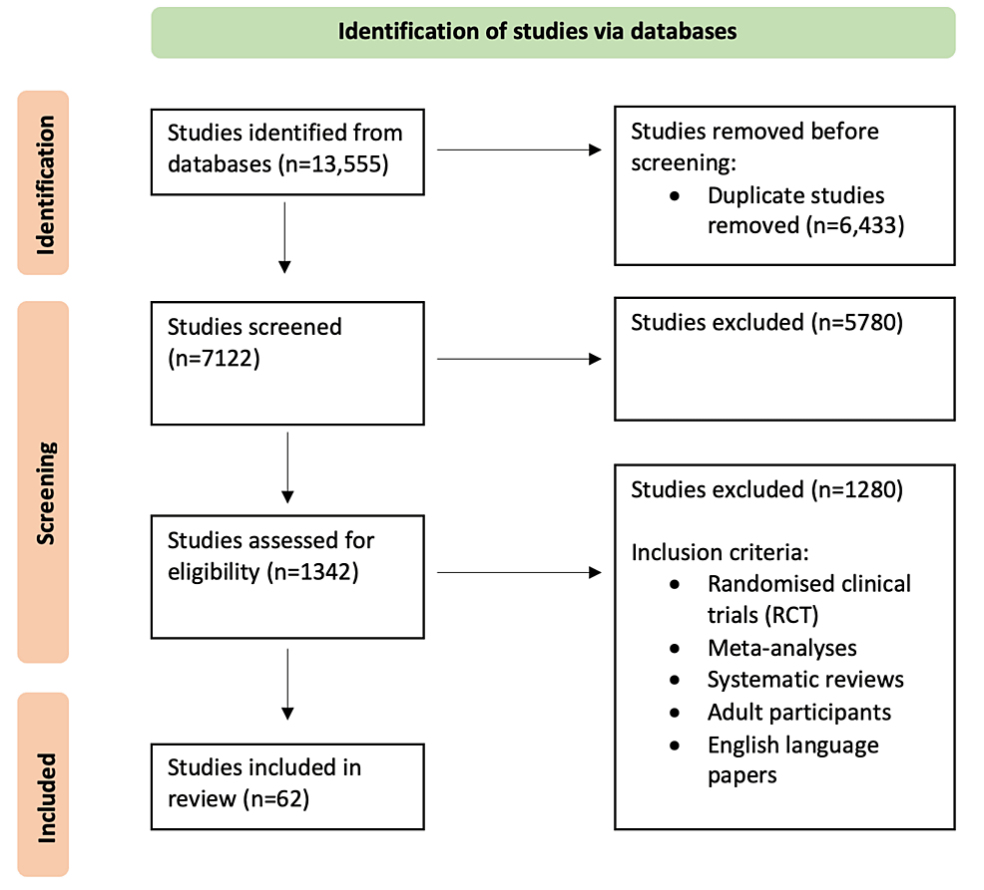
PRISMA flowchart of search methodology. PRISMA: Preferred Reporting Items for Systemic Reviews and Meta-Analysis

The inclusion criteria for publications were randomised clinical trials (RCT), meta-analyses, systematic reviews, adult participants and English language papers. This resulted in a total of 62 studies being included in this literature review. The Preferred Reporting Items for Systemic Reviews and Meta-Analyses (PRISMA) flowchart for the search methodology is shown in Figure 2.

Anti-TNFα drugs

*Infliximab *(*Remicade*®)

The randomised, double-blinded, ACT-1 and ACT-2 trials were one of the first to prove the efficacy of infliximab compared to placebo in moderate-to-severe UC patients [6]. These are highly powered trials with 95% statistical power. In ACT-1, clinical response at week-8 was significantly higher in the infliximab group compared to placebo [6]. Similar findings were observed in ACT-2. Mucosal healing was found to be significantly improved in the infliximab group, a crucial finding as mucosal healing has been shown to be most effective predictor of reduced risk of cancer in UC patients and reduced hospital admissions [6]. In both ACT trials, no significant differences were found between the efficacy of the two doses administered. Thus 5mg/kg is the preferred initial dose of infliximab based on safety and pharmacoeconomic issues [6]. Real-world data also supports the use of infliximab in UC patients [7-8].

A post-hoc analysis of three clinical trials on biological-naïve UC patients by Narula et al. found both infliximab and vedolizumab to have similar efficacy in inducing clinical symptom improvement, though the safety profile of vedolizumab was more favourable [9]. However, infliximab had higher rates of corticosteroid-free clinical remission (CR) and endoscopic remission (ER), suggesting it as the preferential agent for biologic-naïve UC patients [9]. Corticosteroid-free CR is an important outcome as long-term corticosteroid use is linked with serious adverse events (AE), hence higher rates of corticosteroid-free CR provides greater confidence in the effectiveness of the biologic [9]. However, this observed difference between the two drugs may be confounded by the differences in corticosteroid tapering between the trials analysed. Moreover, other differences in the methodology of the trials make it difficult to draw direct comparisons and limit the validity of the findings. Patel et al. [10] evaluated real-world data on the use of vedolizumab and infliximab in biologic-naïve UC patients and found conflicting results to Narula et al. with those receiving vedolizumab having greater long-term real-world effectiveness. However, safety was not assessed in this study [10]. Thus, it is important that head-to-head RCTs comparing the efficacy of the two drugs are performed prior to one being deemed preferential. 

The randomised, double-blinded UC-SUCCESS Trial found combination therapy with azathioprine, an immunosuppressant, and infliximab to be superior to monotherapy with either drug [11]. Corticosteroid-free CR, and mucosal healing were both significantly higher in the combined therapy group 11 (Table 1). However, the early termination of the study resulted in a smaller than expected sample size, reducing the statistical power. A systematic review by Christophorou et al. also found combination therapy with infliximab and immunosuppressants to be superior to infliximab alone in achieving and maintaining CR at four to six months (p<0.01) [12]. However, at 12 months, no significant differences were observed between the two groups (p=0.41), hence combination therapy with immunosuppressants may only have short-term efficacy [12]. 

A summary of the trials mentioned above evaluating infliximab are shown in Table 1.

**Table 1:** Summary of the trials comparing the efficacy of infliximab alongside key findings.

Author	Population size	Comparator	Trial Type	Results
Rutgeerts et al. 2005 (ACT 1) [6]	364 (121 v 243)	Placebo	Randomised, double-blind, placebo-controlled, multicentre trial	Week 8 clinical response was 69% with 5mg infliximab vs 37% with placebo (p<0.001). Week 30 clinical response was 63% with 5mg infliximab vs 36% with placebo (p<0.001). Week 54 clinical response was 45% with 5mg infliximab vs 20% with placebo (p<0.001). The serious adverse event rate was 21.5% with 5mg infliximab vs 25.6% with placebo.
Rutgeerts et al. 2005 (ACT 2) [6]	364 (123 v 241)	Placebo	Randomised, double-blind, placebo-controlled, multicentre trial	Week 8 clinical response was 64% with 5mg infliximab vs 29% with placebo (p<0.001). Week 30 clinical response was 57% with 5mg infliximab vs 32% in with placebo (p<0.001). The serious adverse event rate was 10.7% with 5mg infliximab vs 19.5% with placebo.
Panaccione et al. 2014 (UC SUCCESS) [11]	239	N/A	Randomised, double-blind, double-dummy, multicentre trial	Week 16 corticosteroid-free clinical remission was 39.7% with infliximab/azathioprine vs 22.1% with infliximab alone (p=0.017) and 23.7% with azathioprine alone (p=0.032). Week 16 mucosal healing was 62.8% with infliximab/azathioprine vs 54.6% with infliximab (p=0.295) and 36.8% with azathioprine (p=0.001).

In many UC patients there is loss of response (LOR) to infliximab over time. This was observed in the ACT-1 trial with clinical response reducing from 69% (week-8) to 45% (week-54) [6]. Moreover, in a study by Yamada et al. 72.7% of patients achieved CR but 70.8% then relapsed [13]. However, following infliximab dose intensification 66.7% continued to maintain long-term CR [13]. This study was limited as it only included analysis from a single centre and had a small sample size. Hence, multivariate analysis was unable to be performed to help predict factors influencing the need for dose intensification. However, several other studies have also observed similar results with infliximab dose intensification being required in a significant number of UC patients to maintain CR [14-15].

Therapeutic drug monitoring (TDM) may also be beneficial in individuals with LOR to biologics, infliximab in particular [16-18]. It can help identify the mechanism for the LOR and consequently determine whether dose intensification is required or whether a switch in therapy is more beneficial [17]. TDM is also cost-effective as it can help identify unnecessary use of infliximab, observed in 30.6% of patients in a recent study by Wu et al. [18]. However, multiple barriers exist prior to TDM being used in day-to-day clinical practice, including, the cost of TDM implementation as well as standardisation in the interpretation of results [18].

*Adalimumab *(*Humira*®)

Adalimumab is a safe and effective treatment for inducing and maintaining remission in moderate-to-severe UC patients with both short-term and long-term efficacy [19-21]. The multicentre, double-blind, placebo-controlled ULTRA trials 1 [19], 2 [20] and the open-label extension ULTRA-3 found adalimumab to have maintained rates of remission (63.6%), mucosal healing (59.9%) and improved QOL on up to 4-years of therapy [21]. A summary of these trials is shown in Table 2.

**Table 2:** Summary of trials demonstrating the efficacy of adalimumab alongside key findings.

Author	Population size	Comparator	Trial Type	Results
Colombel et al. 2014 (ULTRA 3 study) [21]	588	N/A	208-week open-label observational study	Up to 3-years remission was 63.6% and mucosal healing was 59.9%
Sandborn et al. 2012 (ULTRA 2 study) [20]	518 (258 v 260)	Placebo	Phase 3, 52-week multicentre, randomised, double-blind, placebo-controlled study	Adalimumab vs placebo clinical response (30.2% v 18.3%), clinical remission at 8 weeks (16.5% v 9.3%), clinical remission at 52 weeks (17.3% v 8.5%), greater rates of remission in anti-TNF naïve patients vs anti-TNF experienced patients at 8 weeks (21.3% v 9.2%), and at 52 weeks (22% v 10.2%) (p<0.05).
Reinisch et al. 2011 (ULTRA 1 study) [19]	575 (353 v 222)	Placebo	Phase 3, 8-week multicentre, randomised, double-blind, placebo-controlled study	Adalimumab vs placebo steroid-free clinical remission (16.9% v 9%). No adverse events of special interest (e.g. tuberculosis, demyelination) or deaths(p<0.05).

Real-world data supported the efficacy of adalimumab often showing improved performance [22-23]. This could be explained by varying reported outcomes and patients requiring dose-escalation being classed as treatment responders. The ULTRA trials were limited by protocol design leading to patients receiving doses at different times. Therefore, to assess long-term efficacy using the same treatment duration only a smaller subset of patients was included (55%) [21]. This reduced the power of the long-term efficacy findings. Furthermore, data analysed from ULTRA-3 only included patients who successfully completed the prior 1-year study limiting its reflection of real-world patients. The open-label nature of the study may have also influenced clinical outcomes. On the other hand, the study had regular follow-ups and assessed various end points to comprehensively evaluate adalimumab’s patient impact.

Comparing treatment efficacy of adalimumab to infliximab from the similarly designed ULTRA and ACT trials found lower remission rates in ULTRA-1 and 2 compared to those found in ACT-1 and 2 but mucosal healing was greater [6,19,20]. The lower ULTRA remission rates could be explained by 40% of patients having previous infliximab treatment, whereas in ACT all patients were anti-TNF naïve. ACT was conducted 10 years before ULTRA, when those refractory to standard treatment had no other available pharmacotherapy. ULTRA made use of rescue therapy, which was not utilised in ACT trials, and used remission as its primary end point whereas ACT used treatment response. Therefore, head-to-head trials are needed to accurately conclude which therapy is superior. 

In ULTRA-2, significantly greater rates of CR were found in anti-TNF naïve patients at 8 and 52 weeks, this was also supported by real-world data [24-25]. Interestingly, Hussey et al. found better outcomes in anti-TNF experienced patients, however, this represented a small subgroup of patients with differing treatment dosages [26]. Furthermore, where LOR was found in adalimumab patients, dose escalation was found to be an appropriate measure to enable recovery of response, with lower rates of dose escalation required in anti-TNF naïve patients [25]. 

The benefit of combined immunomodulator therapy over adalimumab monotherapy remains controversial. A meta-analysis of placebo-controlled trials found adalimumab was safe and effective at doses of 160/80/40mg, although patients receiving additional immunomodulator therapy had superior short-term efficacy [27]. This finding should be treated with caution as it reflects only a small number of low powered studies. Colombel et al. contradicts these findings where no advantage with combined therapy was observed over adalimumab monotherapy. However, monotherapy and combined treatment arms were not randomly assigned, instead were determined by baseline immunomodulator use. Therefore, differing characteristics of the two treatment arms may have confounded comparisons [28]. Hence, double-blinded trials are needed to confirm the benefit of combined therapy.

Real-world data suggests CR, treatment persistence and 1-year colectomy-free survival is comparable between both adalimumab and golimumab [29]. This is supported by propensity score analysis which found no significant difference between adalimumab and golimumab in effectiveness [30-31], yet adalimumab had a reduced risk of treatment discontinuation (hazard ratio {HR}: 0.71) [31]. However, such propensity score analyses are limited by unmeasured confounding factors and hence biased results, reducing external validity. Nevertheless, Gendelman et al. found encouraging rates of persistence and adherence for adalimumab, but concomitant corticosteroid use was associated with an increased risk of treatment discontinuation [32]. Therefore, further studies are required to compare treatment persistence accurately.

*Golimumab *(*Simponi*®)

Golimumab is another safe and effective treatment for moderate-to-severe UC [33-34]. The phase-3 double-blinded PURSUIT-SC trial found subcutaneous golimumab effectively induced CR, mucosal healing, and increased QOL at six weeks compared to placebo in anti-TNF naïve patients [33]. The efficacy and safety of golimumab was also highlighted in the GO-COLITIS trial [34]. However, early induction response was greater and sustained response was lower compared to the PURSUIT trial [34]. This could be explained by differences in study design and in the frequency of clinic follow-ups between the trials. 

All patients in the PURSUIT trial were anti-TNF naïve [33]. There is growing evidence of inferior response to biologic therapy in biologic-experienced patients [35]. In clinical settings, patients are likely exposed to previous biologic therapy. Therefore, this data may not be reproducible in a real-world setting. Thus, similar large trials in anti-TNF experienced patients are needed. Real-world data from a small study found only 14% of patients had complete CR, although 52% of patients in this study were anti-TNF experienced in contrast to the PURSUIT trial [36]. In a small prospective cohort study, CR was achieved in 70% of patients of which 73% were anti-TNF experienced [37]. Taxonera et al. found CR rates were lower if golimumab was administered as a third anti-TNF treatment. Although, at one year 42% of patients had golimumab treatment failure mainly due to primary non-response [38].

The PURSUIT-M trial found remission and response rates were maintained at 30 and 54 weeks of treatment [39]. Recently, the long-term extension (LTE) of PURSUIT-M showed maintained clinical benefit at up to 3 additional years, where 63% of patients remained on golimumab treatment after 228 weeks [40]. However, all patients included in the LTE had: an observed prior clinical benefit in the PURSUIT-M study, 40% treatment discontinuation rates from week 104 and a lower number of placebo patients with a shorter follow up [40]. This reduced the reliability and generalisability of these findings. The relatively high-powered GO-COLITIS trial found sustained CR in only 25% of patients at one year [34], although early assessment at six weeks may have excluded late responders from passing into the maintenance study phase [34]. Surprisingly, 60% of patients who had a sustained CR discontinued treatment, yet still retained a CR for an additional 12 weeks [34]. There is paucity of real-world data on golimumab persistence beyond 2-years [41-42]. Although recently Iborra et al.demonstrated long-term efficacy and safety profiles on up to 4-years of treatment [43]. The chart review design of this study reduced recall bias, however, this also led to missing data and subjective interpretation.

The PURSUIT trial found high golimumab serum concentrations were associated with increased response and remission rates suggesting an exposure-response relationship [33], this was also reflected by real-world data [30,36,43]. The open-label phase 4 GO-LEVEL trial found serum concentrations of 3.8μg/mL at week-6 and 2.4μg/mL during maintenance to be optimal therapeutic thresholds [44]. This study included the largest published prospective cohort of patients undergoing pharmacokinetic monitoring in induction and early maintenance [44]. However, this target was optimised for CR not ER, of which a smaller study found a much higher serum threshold of 7.4μg/mL [45]. Although, undefined normal parameters and inadequate evidence for its implementation mean TDM for golimumab dose optimisation is not yet routinely used in clinical practice.

A summary of the trials mentioned above evaluating the efficacy of golimumab are shown in Table 3.

**Table 3:** Summary of trials demonstrating the efficacy and dose-optimisation of golimumab therapy alongside key findings.

Author	Population size	Comparator	Trial Type	Results
Samaan et al. 2020 (GO-LEVEL study) [44]	102	N/A	Phase 4, open-label, induction: prospective cohort, maintenance: cross-sectional cohort	Induction: golimumab serum concentration, combined clinical and therapeutic remission (5.0 vs 3.1μg/mL) (p<0.05). Receiver operating characteristic (ROC) curve = 3.8μg/mL as the optimal cut-off. Maintenance: serum golimumab concentration, combined remission was significantly higher (2.9 vs 2.1 μg/mL) (p<0.05). ROC curve analysis = 2.4μg/mL as the optimal cut-off.
Probert et al. 2018 (GO-COLITIS study) [34]	205	N/A	Phase 4, open-label, multicentre, single-arm study	Induction: 6 weeks response (68.8%), remission (38.5%). Maintenance: those with clinical response continued into the maintenance phase, clinical remission rates were maintained in 25% of patients at 1-year, improvement in quality-of-life measure (p<0.0001).
Reinisch et al. 2018 (PURSUIT-LTE) [40]	666 (570 v 93)	Placebo	Multicentre, placebo-controlled trial	No frequent adverse events of special interest (including tuberculosis, demyelination or malignancy). At week 228 anti-drug antibody rates with golimumab 50 mg and 100 mg were 4.4% and 3.7%, respectively. At week 216 in induction responders no or mild disease activity (99.3%), corticosteroid-free (92.5%), IBDQ score of ≥170 (76.1%).
Sandborn et al. 2014 (PURSUIT-M) [39]	1399 (1114 v 285)	Placebo	Phase 3, double-blind, randomised trial.	Clinical response at week 54 on 50mg vs 100mg golimumab vs placebo (47% vs 49.7% vs 31.2%)(p<0.01). At week 30 and 54 on 100mg golimumab vs placebo clinical remission (27.8% vs 15.6%), mucosal healing (42.4% vs 26.6%) (p<0.05). 50 mg clinical remission (23.2% vs 15.6%), mucosal healing (41.7% vs 26.6%). Serious adverse events placebo vs 50mg vs 100mg (7.7% v 8.4% v 14.3%) and serious infection (1.9% v 3.2% v 3.2%).
Sandborn et al. 2014 (PURSUIT-SC) [33]	1395 (1064 v 331)	Placebo	Integrated double-blind phase 2 and phase 3 trial	At Week 6 clinical response 200mg/100mg (51.0%), 400mg/200mg (54.9%) vs placebo (30.3%) (p<0.0001). Clinical remission and mucosal healing significantly higher in golimumab vs placebo arms (p<0.005). Serious adverse events placebo vs golimumab (6.1% v 3%) and serious infection (1.8% v 0.5%).

LOR is a major concern for all anti-TNF treatment and dose escalation can be a way to overcome this. However, it is not yet widely licensed for golimumab as it is for adalimumab and infliximab. Notwithstanding this, golimumab dose escalation has been shown to recover response significantly [38,46]. Therefore, LOR and reduced therapeutic dosing is a barrier to optimising golimumab treatment efficacy.

Anti-integrin drugs

*Vedolizumab *(*Entyvio*®)

Many trials have shown vedolizumab to be superior to placebo in the treatment of UC [47-49]. The first significant study was the GEMINII study, demonstrating greater response rate and CR in vedolizumab compared with placebo [47]. These results were further supported by a Cochrane meta-analysis using four studies, demonstrating that vedolizumab was better than placebo in achieving CR and ER [50].

Despite the GEMINII study being very well-designed, there were some limitations. Approximately 80% of the patients were Caucasian in both the vedolizumab and placebo cohorts. The efficacy of vedolizumab may not be representable in other ethnicities such as Asians, especially when the incidence of UC is rising in Asian countries [51]. Furthermore, a greater proportion of patients on placebo had failed previous anti-TNF therapy, suggesting that these patients already had a worse prognosis than the vedolizumab group, allowing for a greater difference in results. Nevertheless, vedolizumab has been shown to be very effective in treating UC in the real-world setting, achieving good clinical response and high CR rates [52].

Even though there is a lack of direct comparison between different interventions in the treatment of UC, the VARSITY trial compared adalimumab with vedolizumab, demonstrating greater CR in vedolizumab compared with adalimumab (31.3% vs 22.5%, p=0.006) [53]. Vedolizumab had a lower failure rate and higher discontinuation-free survival than adalimumab in infliximab-failed patients in a real-world setting [54]. 

However, patients who previously had no response to anti-TNF therapy were eligible for the VARSITY trial, potentially lowering the overall efficacy of adalimumab. Furthermore, dose escalation was not permitted in this study, even though dose escalation of adalimumab is known to induce CR in patients who have had no response. For example, Taxonera et al. demonstrated that dose escalation of adalimumab allowed 47% of patients who have had no response to achieve CR [25]. Therefore, the dosage of adalimumab may have been too low.

The EVOLVE study found that vedolizumab had similar clinical response rates, remission rates and mucosal healing when compared with anti-TNF therapy, which included adalimumab, golimumab and infliximab, in a real-world setting [55]. Patients on vedolizumab had significantly fewer AEs than anti-TNF therapy, higher patient persistence and required less dose escalation. 

As this was a retrospective study, there will be missing data and possible bias introduced when analysing the data. The anti-TNF cohort also had significantly fewer patients than the vedolizumab cohort, reducing the power of the study. A summary of trials mentioned comparing vedolizumab with placebo or other drugs are shown in Table 4.

**Table 4:** Summary of trials demonstrating the efficacy of vedolizumab alongside key findings.

Author	Population size	Comparator	Trial Type	Results
Bressler et al. 2021 (EVOLVE Study) [55]	604 (380 v 224)	Anti-TNF drugs (adalimumab, golimumab and infliximab)	Multinational, multicentre retrospective trial	Similar clinical remission rates, clinical response rates and mucosal healing Vedolizumab had less adverse events than anti-TNF drugs (HR 0.37 v 0.56). Higher patient persistence was observed in the vedolizumab cohort at 24 months (76.3% v 52.4%).
Sandborn et al. 2020 [48]	162 (106 v 56)	Placebo	Phase 3, double blind, double dummy, multicentre and international trial	Higher clinical remission at week 52 in vedolizumab vs placebo (46.2% v 14.3%). Greater endoscopic improvement (p<0.001).
Favale et al. 2019 [54]	161 (97 v 64)	Adalimumab	Retrospective, multicentre and real-life study	Adalimumab had a higher failure rate than vedolizumab in infliximab-failed patients (60% v 28.9%). Discontinuation-free survival was higher in patients on vedolizumab than adalimumab (319 days v 251 days).
Sands et al. 2019 (VARSITY trial) [53]	769 (383 v 386)	Adalimumab	Phase 3b, double blind, double dummy, randomised multicentre and international trial	Higher clinical remission at week 52 in vedolizumab vs adalimumab patients (31.3% v 22.5%). Histologic remission higher in vedolizumab at week 52 (10.4% v 3.1%).
Motoya et al. 2019 [49]	292 (164 v 82)	Placebo	Phase 3, double blind, randomised, placebo-controlled study	No significant difference between vedolizumab and placebo as induction therapy at week 10 (p=0.2722). Higher clinical remission in vedolizumab vs placebo at week 60 (56.1% v 31.0%).
Feagan et al. 2013 (GEMINI I study) [47]	474 (225 v 149)	Placebo	Phase 3, double blind, randomised, placebo-controlled, multicentre and international study	Greater response rate in vedolizumab at week 6 (47.1% vs 25.5%). Higher clinical remission in vedolizumab Q4W vs placebo (44.8% vs 15.9%). Higher clinical remission in vedolizumab Q8W vs placebo (41.8% vs 15.9%).

One main concern of vedolizumab are the potential AEs. Natalizumab, also an α4 integrin inhibitor, increased the risk of developing progressive multifocal leukoencephalopathy (PML) [56], and was taken off the market in 2005. The GEMINI long-term safety study (GEMINI LTS) by Feagan et al. monitored patients on vedolizumab for up to 9-years and found no risks of developing PML [47]. It was concluded that there were no safety concerns using vedolizumab [57]. Danese et al. reported high patient persistence of 93.9% on vedolizumab over two years [58], and Vermiere et al. reported increased patient QOL using the Inflammatory Bowel Disease Questionnaire (IBDQ) [59].

Another critical factor is the cost-effectiveness of vedolizumab. Vedolizumab is an expensive drug as it is used as maintenance therapy, therefore patients will be required to take vedolizumab for many years, and the GEMINI LTS study reported patients who were on vedolizumab for up to nine years [57]. However, when calculating costs with quality-adjusted life years (QALY), Wilson et al. calculated that vedolizumab was the most cost-effective and associated with the highest QALY compared to other forms of biological treatments [60]. 

Anti-IL-12/23 drugs

*Ustekinumab *(*Stelara*®​​​​​​​)

The UNIFI study compared various doses of ustekinumab with placebo in patients with moderate-to-severe UC who either had an inadequate response or unacceptable AEs to anti-TNFs, vedolizumab or conventional therapies [61]. The study consisted of an induction trial and a maintenance trial. Results showed that those receiving ustekinumab were more likely to achieve CR compared to placebo at the end of both the induction trial and maintenance trial 61 (see Table 5). However, these percentages should be considered with the awareness that those who did not respond to ustekinumab in the induction trial were not entered into the maintenance trial. Had they completed this, the CR for ustekinumab would most likely have been different. However, the study was strengthened through its double-blinding and randomisation, thereby reducing observer and selection bias. It also had a large sample size (961) representing patients internationally (244 sites), improving the validity of the trials [61].

**Table 5:** Summary of trials demonstrating the efficacy of ustekinumab alongside key findings.

Author	Population size	Comparator	Trial Type	Results
Sands et al. 2019 (UNIFI Study) [61]	961 (642 v 319)	Placebo	Phase 3, double blind, multicentre, randomised controlled trial	Those receiving ustekinumab are more likely to achieve clinical remission than those receiving placebo at end of both induction trial (15.6%/15.5% vs 5.3%, p<0.001 for both comparisons) and maintenance trial (38.4%/43.8% vs 24%, p=0.002 and p<0.001 respectively).
Ochsenkühn et al. 2020 [62]	19	N/A	Retrospective cohort study	53% of patients achieved clinical remission after one year of ustekinumab therapy.
Fumery et al. 2021 [63]	103	N/A	Retrospective cohort study	32% of patients achieved clinical remission after one year of ustekinumab therapy.

Smaller studies have also investigated the efficacy of ustekinumab. Two retrospective cohort studies showed similar results to the UNIFI study [62-63], whereby 53% [62] and 32% [63], respectively of patients achieved CR after one year of ustekinumab therapy. However, these studies have very small sample sizes (19 and 103 patients, respectively), reducing their validity. Other limitations which must be considered include their lack of blinding, and the patients studied by Ochsenkühn et al. were still under the influence of vedolizumab whilst starting ustekinumab [62], which may reduce validity of the results. A retrospective cohort study showed that dose intensification is effective in patients who responded poorly to standard doses of ustekinumab, with 55% of patients achieving CR 12-16 weeks after dose escalation [64]. However, this study had a small sample size (108 patients with only 46 requiring intensification), and there is currently no evidence whether dose intensification influences long-term CR. 

It is unclear as to why some patients have a poor response to ustekinumab. However, a meta-analysis of eight RCTs highlighted that those who previously responded poorly to other biological therapy generally had an inferior response to ustekinumab than other patients [35]. This could be an interesting development in further research on the mechanisms behind the lack of responsiveness to biological therapies in some patients.

The safety of ustekinumab was documented in the UNIFI study with reports of several AEs, the commonest being headache. In addition, three deaths occurred, and seven people were diagnosed with various types of cancer [61]. However, a meta-analysis of 30 RCTs found that there was no statistically significant difference in AEs with the use of ustekinumab in inflammatory bowel disease patients compared to other biological therapies (p>0.05 for all) [65]. However, the lack of ustekinumab dose variability and short follow-up time must be considered, which may have caused late-occurring AEs to be missed. 

There appears to be a lack of literature directly comparing ustekinumab to other biologic therapies, however, a meta-analysis of 39 studies showed ustekinumab to have a higher Bayesian probability of clinical response, CR and improved endoscopic-mucosal healing after one year of treatment compared with other licensed biological therapies [1]. However, there have been no head-to-head studies between ustekinumab and other biological agents, making it difficult to understand the appropriate situation in which to give a UC patient ustekinumab.

## Conclusions

This review discusses the efficacy and safety of different biologics. Due to the long-term and systemic AEs of corticosteroids, biologics may be the preferred drug in the future for acute treatment of UC. Although aminosalicylates will most likely remain first-line treatment for maintaining remission, this review hopes to increase the awareness of different biologics and emphasise the importance of comparing different biological and non-biological treatments to determine the optimal treatment plan for patients.

This review utilised high-powered seminal studies and real-world data to reflect the translation of study findings into clinical practice. It used multiple search databases, reflecting the scope of current evidence. However, as with all literature reviews, not all relevant literature could be included. This review was also not inclusive of all patient groups as paediatric and obstetric patients were excluded, thus future reviews on these populations are necessary. Furthermore, biosimilars were beyond the scope of this paper but represent another exciting focus for future reviews.

As the patents on Remicade® have expired, biosimilars and biobetters have been introduced, reducing costs and increasing the availability of this drug to patients. The patents of Entyvio®, Simponi®, Humira® and Stelara® are also expiring soon, which may increase the development of biosimilars in the treatment of UC, and therefore allow more studies to be performed comparing the efficacies of these drugs. Furthermore, other administration routes of these drugs can be studied, such as per rectum (PR) administration, which may increase the bioavailability at the preferred sites in the colon.
